# Supplementary material for: Digitally virtualized atoms for acoustic metamaterials
Source: Nat Commun. 2020 Jan 14;11:251. doi: 10.1038/s41467-019-14124-y (PMC6959268; doi:10.1038/s41467-019-14124-y)
Supplement: Supplementary file 1 — Supplementary Information [file 41467_2019_14124_MOESM1_ESM.pdf]

Supplementary Information for
Digitally virtualized atoms for acoustic metamaterials

Choonlae Cho[1,2†], Xinhua Wen[1†], Namkyoo Park[2], Jensen Li[1*]

Correspondence to: jensenli@ust.hk

Supplementary Note 1. Monopolar and dipolar model of the virtualized atom

For a one-dimensional (1D) system (along *x*-direction), we express the total pressure and the velocity (in the propagating direction in *x*) by

$$p = a_0 \cos(kx) + a_1 i \sin(kx) + b_0\, e^{ik|x|} + b_1 \mathrm{sgn}(x) e^{ik|x|},$$
$$\rho c v_x = a_0 i \sin(kx) + a_1 \cos(kx) + b_0\, \mathrm{sgn}(x) e^{ik|x|} + b_1 e^{ik|x|}, \tag{1}$$

where p and v_x are the pressure and velocity fields respectively, ρ and c are the density and the sound speed in air. The monopolar (dipolar) incident waves are denoted by a_0 (a_1) while the monopolar (dipolar) scattered waves are denoted by b_0 (b_1) in generating symmetric (antisymmetric) waves, see Supplementary Fig. 1 for the schematic representation. The response of the artificial atom can be denoted by the scattering matrix $[\mathcal{D}]$:

$$\begin{pmatrix} b_0 \\ b_1 \end{pmatrix} = [\mathcal{D}] \begin{pmatrix} a_0 \\ a_1 \end{pmatrix} = \begin{pmatrix} \mathcal{D}_{00} & \mathcal{D}_{01} \\ \mathcal{D}_{10} & \mathcal{D}_{11} \end{pmatrix} \begin{pmatrix} a_0 \\ a_1 \end{pmatrix}, \tag{2}$$

which can also be written in terms of transmission (t) and reflection (r) coefficients in the forward (subscript f) and backward (subscript b) directions:

$$[\mathcal{D}] = \frac{1}{4} \begin{pmatrix} -2 + r_b + r_f + t_b + t_f & -r_b + r_f - t_b + t_f \\ r_b - r_f - t_b + t_f & -2 - r_b - r_f + t_b + t_f \end{pmatrix}. \tag{3}$$

The response of the system can be equivalently described by $[Y](\omega)$ matrix for convolution operation (in Fig. 1b) or the scattering matrix $[\mathcal{D}](\omega)$. They are related to each other by

$$\begin{pmatrix} M_1 \\ M_2 \end{pmatrix} = [Y]^{-1} \begin{pmatrix} S_1 \\ S_2 \end{pmatrix} = \begin{pmatrix} \cos\phi & -i \sin\phi \\ \cos\phi & i \sin\phi \end{pmatrix} [\mathcal{D}]^{-1} \begin{pmatrix} \cos\delta & \cos\delta \\ i \sin\delta & -i \sin\delta \end{pmatrix} \begin{pmatrix} S_1 \\ S_2 \end{pmatrix} + [G] \begin{pmatrix} S_1 \\ S_2 \end{pmatrix}. \tag{4}$$

where $[G]$ is the structure factor in describing the propagation from two speakers S_1, S_2 to microphones M_1, M_2 and is defined by

$$[G] = e^{i\phi} \begin{pmatrix} e^{-i\delta} & e^{i\delta} \\ e^{i\delta} & e^{-i\delta} \end{pmatrix}. \tag{5}$$

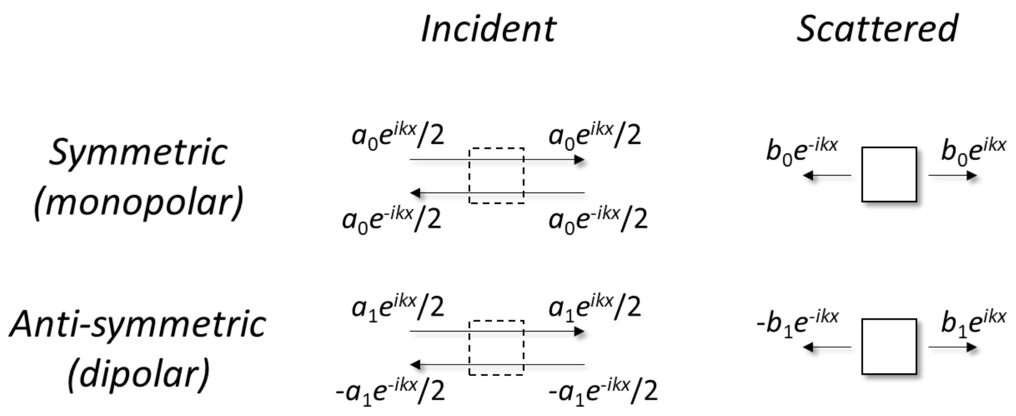

Supplementary Figure 1 | The schematic diagram for monopolar and dipolar models of the virtualized atom. The scattering process is defined as a monopolar response when the symmetric input generates the symmetric scattering field (upper). On the contrary, an anti-symmetric input generates an anti-symmetric scattering field (lower) is defined as a dipolar response. Here $a_{0,1}$ and $b_{0,1}$ represent amplitudes of the incident and scattered waves, respectively. The center of the virtualized atom is set at the origin.

where 2ϕ is the phase distance between two microphones and 2δ is the phase distance between two speakers (see Fig. 1b). $[G]$ modifies the response matrix $[Y]$ since microphones detect incoming waves plus the secondary radiation generated by speakers. Such a "renormalization" from the circuit response to the overall atomic response is common. It also occurs in the effective medium theory for an ensemble of isolated dipoles: the macroscopic and local fields are different so that susceptibility is proportional to polarizability in the dilute limit but goes to the Maxwell Garnett formula as filling fraction increases due to a similar renormalization.

From the relationship between $[\mathcal{D}]$ and $[Y]$, we can solve $[\mathcal{D}]$ from given $[Y]$. For example, the scattering matrix for the monopolar atom in Figs. 2 and 3 can be obtained by substituting $Y_{11} = Y_{12} = Y_{21} = Y_{22} = Y/2$ to obtain

$$[\mathcal{D}](\omega) = \begin{pmatrix} \mathcal{D}_{00}(\omega) & \mathcal{D}_{01}(\omega) \\ \mathcal{D}_{10}(\omega) & \mathcal{D}_{11}(\omega) \end{pmatrix} = \frac{2Y(\omega)}{\sec\delta - 2\,Y(\omega)e^{i\phi}} \begin{pmatrix} \cos\phi & 0 \\ 0 & 0 \end{pmatrix}. \quad (6)$$

Similarly, we can also readily obtain a virtualized metamaterials with dipolar scattering response by redesigning the $[Y]$ matrix as $Y_{11} = -Y_{12} = -Y_{21} = Y_{22} = Y/2$. Then the scattering matrix $[\mathcal{D}](\omega)$ becomes

$$[\mathcal{D}](\omega) = \begin{pmatrix} \mathcal{D}_{00}(\omega) & \mathcal{D}_{01}(\omega) \\ \mathcal{D}_{10}(\omega) & \mathcal{D}_{11}(\omega) \end{pmatrix} = \frac{2Y(\omega)}{\csc\delta + 2i\,Y(\omega)e^{i\phi}} \begin{pmatrix} 0 & 0 \\ 0 & \sin\phi \end{pmatrix}. \quad (7)$$

Theoretically, only the dipolar scattering coefficient $\mathcal{D}_{11}$ is nonzero in $[\mathcal{D}]$ matrix, but the discrepancy between two speakers of the atom inevitably generates small amounts of monopolar secondary radiation in the experiment. Therefore, the monopolar scattering coefficient $\mathcal{D}_{00}$

becomes nonzero, but is very small compared to the dipolar scattering coefficient $\mathcal{D}_{11}$, as shown in Fig. 4a,b. In a more conventional way to describe the response of the atom, we can use the polarizability $[\alpha]$, which is related to the $[\mathcal{D}]$ matrix by

$$[\alpha](\omega) = \begin{pmatrix} \alpha_{00}(\omega) & \alpha_{01}(\omega) \\ \alpha_{10}(\omega) & \alpha_{11}(\omega) \end{pmatrix} = \frac{2c}{i\omega}[\mathcal{D}](\omega). \quad (8)$$

Supplementary Note 2. Power gain of active metamaterials

Supplementary Figure 2 plots the power gain $|r|^2 + |t|^2$ for configurations in Fig. 2, confirming the outgoing power is larger than the input power.

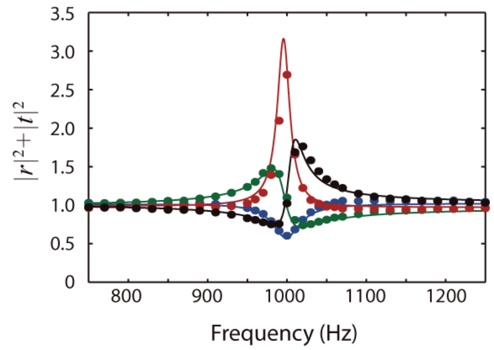

Supplementary Figure 2 | Metamaterial power gain. The spectrum for the sum of transmission intensity and reflection intensity for 4 configurations (in Fig. 2) with convolution phase $\theta = 0°$ (blue), 90° (green), 180° (red) and 270° (black), where symbols and lines denote the experimental and theoretical results respectively. Here $|r|^2 + |t|^2$ is the power gain for one-side incidence.

Supplementary Note 3. Effective medium parameters and impedance matching

As shown in Fig. 4, the monopolar and dipolar response of the virtualized metamaterial can be controlled independently. Therefore, we can readily realize the decoupled control on the effective medium parameters since the system can be regarded as an effective medium, which breaks through those limitations inherent to the physical structures. The spectrum of the effective medium parameters is determined by Eq. (6), as shown in Supplementary Fig. 3. For the virtualized metamaterial with dipolar response only (Supplementary Fig. 3a,b, the monopolar resonance strength $a_0 = 0$), the dipolar susceptibility χ_1 which is associated with the effective mass density ρ, is dominated over the monopolar susceptibility χ_0 for both the case of convolution phase $\theta = 0°$ and 180°. When the monopolar resonance strength a_0 increases from 0 to 6.3 Hz, the monopolar susceptibility χ_0 emerges (Supplementary Fig. 3c,d). The virtualized metamaterial behaves like an ensemble of isolated dipolar and monopolar resonance. Therefore, we can further adjust the dipolar

and monopolar resonance independently to realize the impedance matching. Readjust the resonance strength a_0 to 4.0 Hz, only the monopolar susceptibility χ_0 decreases while the dipolar susceptibility χ_1 keeps the same (Supplementary Fig. 3e,f). In this case, the inverse of effective bulk modulus B^{-1} and the effective mass density ρ are almost equivalent, which represents the realization of impedance matching ($Z = \sqrt{\rho / B^{-1}} \approx 1$). As a result, we can adjust the reflection and transmission amplitudes and achieve small reflectance in a wide frequency regime as shown in Supplementary Fig. 4.

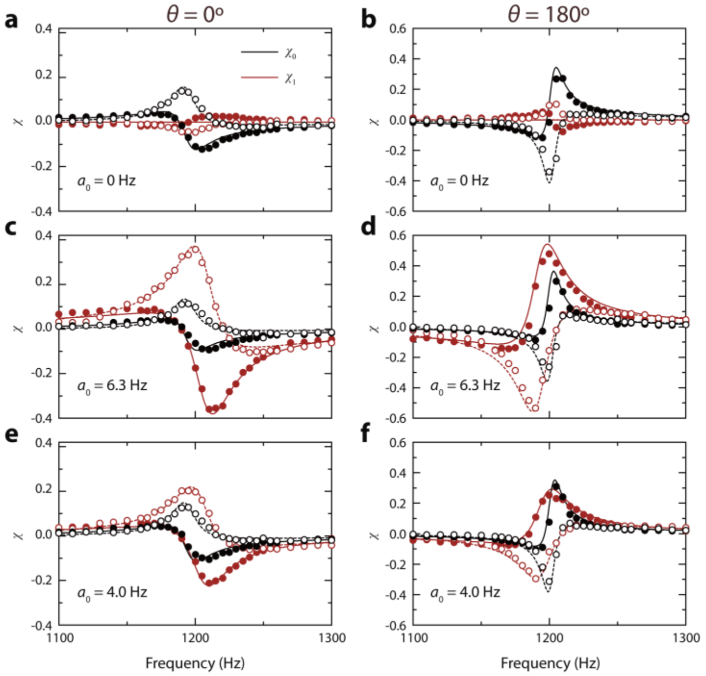

Supplementary Figure 3 | The effective medium parameters of the virtualized metamaterial. The monopolar and dipolar susceptibilities χ_0 (red) and χ_1 (black) for the convolution phase of $\theta = 0°$ and 180°. **a,b,** When the virtualized metamaterial has a dipolar response only, the dipolar susceptibility χ_1, associated with the effective density ρ, is dominated in this case. **c,d,** The monopolar susceptibility χ_0 which is associated with the effective bulk modulus B of the virtualized metamaterial emerges after adding the monopolar response. **e,f,** The monopolar susceptibility χ_0 decreases while χ_1 keeps the same when we lower the resonance strength a_0. For all results, lines and symbols represent the theoretical and the experimental results, respectively. The left and right panels show the scattering coefficients for the convolution phase of $\theta = 0°$ and 180°.

On the other hand, the connection from the single-atom polarizabilities to the bulk property (multiple atoms) can be obtained from Eq. 6. In our case of metamaterial atom in a 1D system, the near field in coupling the neighboring unit cells (along the propagating direction) is not significant. The following simulation shows that the wavefront quickly goes to plane wave within 5 cm, which is comparable to the size of each atom (6.5 cm), and much smaller than the wavelength of acoustic wave (34 cm).

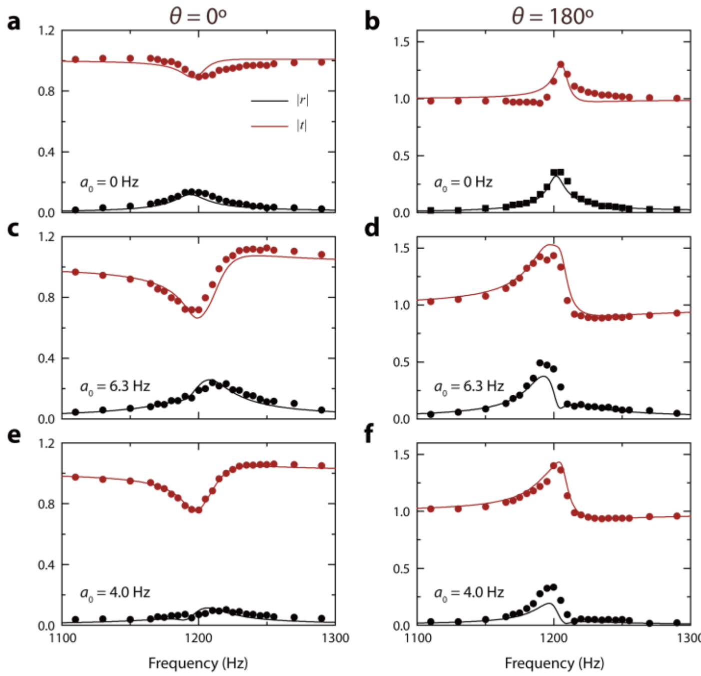

Supplementary Figure 4 | The reflection and transmission amplitudes. The reflection amplitude $|r|$ (black) and transmission amplitudes $|t|$ (red) the convolution phase of θ = 0° and 180°, when the virtualized metamaterial has a dipolar response only (**a,b**), the monopolar response is added (**c,d**), and the monopolar resonance strength a_0 is decreased to realize the impedance matching (**e,f**). For all the cases of convolution phase θ = 180° (right panel), the virtualized atom is working in the active regime and the peak of the transmission amplitudes beyond the unity.

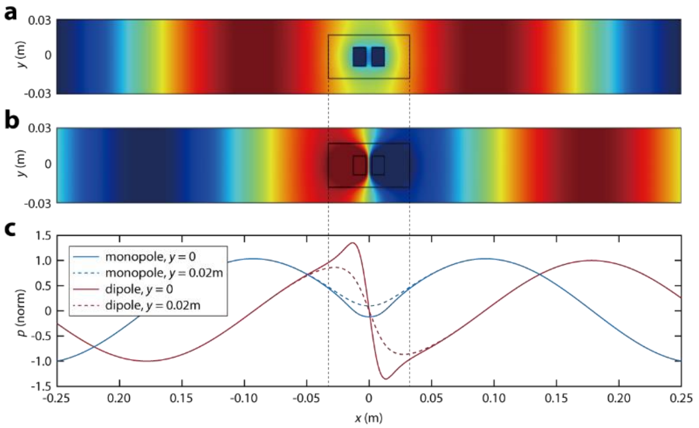

Supplementary Figure 5 | The plane wave generation from meta-atom speakers. a,b, Numerical simulations of meta-atom sources generating (**a**) monopolar (symmetric) and (**b**) dipolar (anti-symmetric) scattering fields. **c,** Pressure field profiles measured at y = 0 (solid line) and y = 0.02 m (dashed line) for monopolar (blue) and dipolar (red) sources, where the wavefront goes to plane wave within 0.05 m. Speakers modeled by 1 × 1.5 cm flat rectangular structure are 1.7 cm away from each other and mounted in 2 × 6 cm rectangular waveguide.

This is in the so-called transmission-line metamaterial regime in which the near-field coupling between neighboring unit cells becomes negligible while the coupling between the neighboring unit cells is only through far-field. With this background, the single atom property also represents the bulk property when atoms are cascaded in the propagating direction. Supplementary Figure 6a shows the more traditional representation of effective medium parameters of the same type of atoms in Fig. 2 (with a smaller resonance strength and convolution phase 180°), now in terms of an anti-Lorentzian resonating reciprocal bulk modulus and a unit density (not shown here). Solid lines/symbols are the extracted model/experimental values, and black/blue colors represents the real/imaginary part. Then, by using these effective single-atom medium properties, we can calculate the expected two-atoms and three-atoms properties, transmission amplitude and phase spectra are shown in Supplementary Fig. 6b,c, as solid lines, which also agree to the experimental results shown in symbols, showing the validity in using single atom property in scaling up to the situation of multiple atoms.

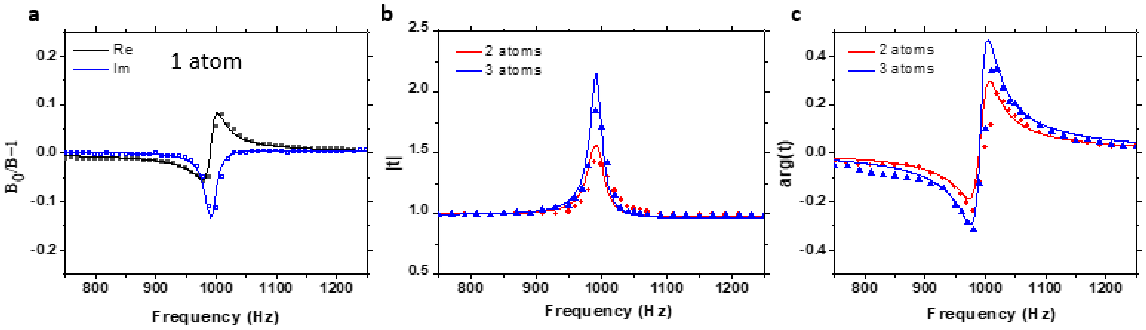

Supplementary Figure 6 | Response from multiple atoms. a, Effective medium parameters extracted from a single atom with convolution phase θ = 180°. Solid lines (symbols) represent theoretical (experimental) results. The real (imaginary) part is shown in black (blue) color. **b,c,** Transmission from cascading 2 or 3 atoms of the same configuration. Experimental results, (**b**) for 2 atoms and (**c**) 3 atoms, are shown in symbols, while solid lines represent the theoretical results that are obtained from the previous single-atom property shown in **a**.

Supplementary Note 4. Transient response of the metamaterial

The response of a resonating metamaterial generally depends on the Q-factor, or equivalently the resonating linewidth, of the resonance. The response time can be measured experimentally by using a step function (with the carrier frequency, e.g., 1 kHz) as input to drive the incident wave. As an example, we shine such an incident wave on the metamaterial (in Fig. 2) with different resonating linewidth γ = 7.5, 15, and 30 Hz. The response time, defined as the time to get half of the steady-state amplitude, is found as 29, 14 and 7 ms, which is roughly proportional to $1/\gamma$.

On the other hand, we can also fire a transient signal with a varying amplitude as a wave packet for the incident wave, instead of getting the monopolar response at separate frequencies by firing continuous waves at different frequencies. Here, the responses from 750 to 1250 Hz are measured

in one single transient experiment by inverse Fourier transforming the measured signal at various microphones. Supplementary Figure 8a shows the input pulse (quadratic spline) with a duration of 5.0 ms and 2.5 ms. The carrier frequency of the pulse is set as 1 kHz. Such an incident transient pulse is fired to the metamaterial, with the configuration in Fig. 2 with convolution phase $\theta = 0°$ and resonating linewidth γ = 15 Hz. Supplementary Figure 8b,c show the corresponding experimental spectrum (solid/empty symbols for real/imaginary part) obtained for the monopolar polarizability. As we can see, the results follow the theoretical spectrum (in lines) very well, even up to a pulse width as short as 5.0 ms, which is much shorter than the response time 14 ms, indicating the metamaterial can work for transient excitations. When the pulse width is further reduced, we see higher noise as the total power of the incident wave is now spread across a wider range of frequencies.

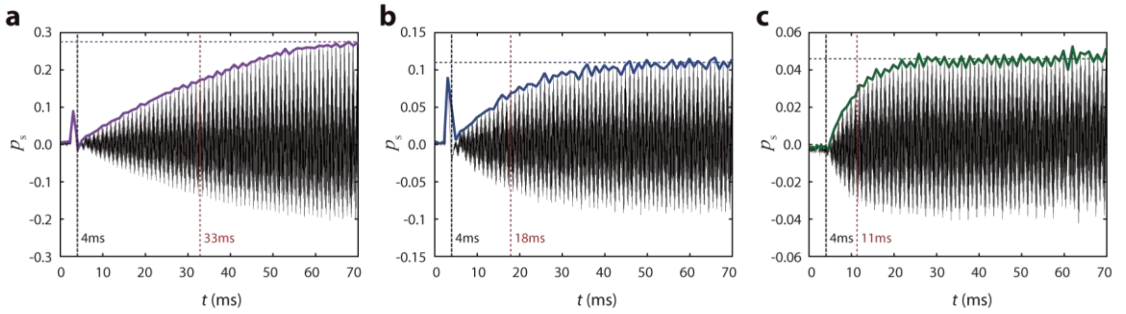

Supplementary Figure 7 | Transient response for step-type input at a fixed frequency. Typical response time to approach steady-state with amplitude response agreeing to target spectrum for (**a**) resonating linewidth γ = 7.5 Hz, (**b**) 15 Hz, and (**c**) 30 Hz for same resonating frequency 1 kHz and the same incident step function with carrier frequency 1 kHz in experiments. Smaller γ has a shorter response time. The time constant for each bandwidth is given by 29 ms, 14 ms, and 7 ms.

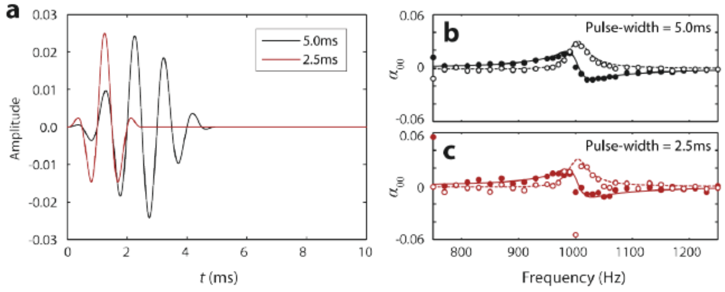

Supplementary Figure 8 | Transient response for finite pulses. a, The input (quadratic spline) pulses with finite duration 5.0 ms or 2.5 ms, and carrier frequency 1 kHz in driving the incident waves. **b,c,** The monopolar polarizability measured from the metamaterial in Fig. 2 with convolution phase $\theta = 0°$ and resonating linewidth γ = 15 Hz.

On the other hand, the response time (due to electronics and digital sampling) can be probed by firing a pulse to our metamaterial but with a non-resonating response (small resonating strength). Supplementary Figure 9 shows a typical incident pulse (in black line) and the measured scattering from the metamaterial (red dashed line). It is found that the response time is around 500 ms, which

includes all the physical and electronic delays between the microphones and speakers with feedback.

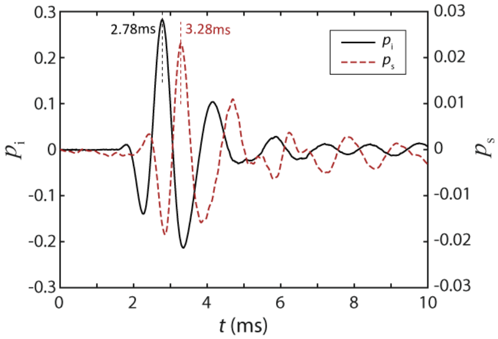

Supplementary Figure 9 | Response time for a non-resonating metamaterial. Incident (p_i) and scattering (p_s) waves of a non-resonating metamaterial in probing the ultimate response time, due to all physical and digital electronic delay.
